# Knowledge of Antimalarials and Health Seeking Behaviour of Households in Case of Suspected Malaria in Democratic Republic of the Congo

**DOI:** 10.3390/tropicalmed6030157

**Published:** 2021-08-26

**Authors:** Nsengi Y. Ntamabyaliro, Christian Burri, Yves N. Lula, Daniel Ishoso, Aline B. Engo, Mireille A. Ngale, Jerry Y. Liwono, Eric S. Mukomena, Gauthier K. Mesia, Samuel M. Mampunza, Gaston L. Tona

**Affiliations:** 1 Unité de Pharmacologie Clinique, Faculté de Médecine, Université de Kinshasa, Avenue de l’Université 1, Kinshasa, Democratic Republic of the Congo; yves.lula@unikin.ac.cd (Y.N.L.); aline.engo@unikin.ac.cd (A.B.E.); ngalemireille@gmail.com (M.A.N.); jerryliwono@yahoo.fr (J.Y.L.); mesia.kahunu@unikin.ac.cd (G.K.M.); mampunza552@gmail.com (S.M.M.); tonalutete@gmail.com (G.L.T.); 2 Swiss Tropical and Public Health Institute, Department of Medicine, Socinstrasse 57, 4002 Basel, Switzerland; christian.burri@swisstph.ch; 3 Department of Pharmaceutical Sciences, University of Basel, Petersplatz 1, 4001 Basel, Switzerland; 4 École de Santé Publique, Faculté de Médecine, Université de Kinshasa, Kinshasa, Democratic Republic of the Congo; ishoso@yahoo.fr; 5 Programme National de Lutte Contre le Paludisme, Ministère de la Santé, Kinshasa, Democratic Republic of the Congo; mukomena3@gmail.com; 6 Faculté de Médecine, Université de Lubumbashi, BP 1825, Lubumbashi, Democratic Republic of the Congo; 7 Centre Neuropsychopathologique, Faculté de Médecine, Université de Kinshasa, Kinshasa, Democratic Republic of the Congo

**Keywords:** households, antimalarial, health seeking behaviour, malaria, malaria treatment

## Abstract

(1) Background: The Democratic Republic of the Congo (DRC) is heavily affected by malaria despite availability of effective treatments. Ignorance and unrecommended behaviour toward a suspected malaria case in households may contribute to this problem. (2) Method: In communities of one rural and one urban Health Centres in each of the 11 previous provinces of DRC, all households with a case of malaria in the 15 days prior to the survey were selected. The patient or caregiver (responder) were interviewed. Logistic regression was used to assess predictors of knowledge of recommended antimalarials and adequate behaviour in case of suspected malaria. (3) Results: 1732 households participated; about 62% (1060/1721) of the responders were informed about antimalarials, 70.1% (742/1059) knew the recommended antimalarials and 58.6% (995/1699) resorted to self-medication. Predictors of knowledge of antimalarials were education to secondary school or university, information from media and smaller households. Predictors of good behaviour were Catholic religion and smaller households. Receiving information from Community Health Workers (CHWs) failed to be determinants of knowledge or adequate behaviour. (4) Conclusion: malaria control in DRC is hampered by ignorance and non-adherence to national recommendations. These aspects are influenced by unsuccessful communication, size of households and level of education.

## 1. Introduction

Malaria is still causing a significant health burden in the Democratic Republic of the Congo (DRC) despite the implementation of numerous disease control tools including potent drugs. Over 40,000 people of the country’s 90 million inhabitants, mainly children under five years old, still die from malaria per year [1]. Numerous parameters influence the effectiveness of drugs in everyday use. One of the under-researched factors negatively influencing effectiveness is the irrational use of drugs in health facilities and communities. The main symptom of malaria is fever. Studies have estimated that children under five years of age suffer from 2 to 11 fever episodes per year [2,3], often from a fever of other origin [3,4]. Given an average household size of five to six members in the DRC [5], and the incidence of malaria and other febrile illnesses, a health seeking decision with potentially fatal consequences has on average to be made every week.

The WHO and the National Malaria Control Program/Programme National de Lutte contre le Paludisme (PNLP) of the DRC recommend that each case of malaria confirmed by a malaria Rapid Diagnostic Test (mRDT) or microscopy shall be treated with recommended drugs as quickly as possible, latest within 24 h [6,7]. Artemisinin-based combination therapies (ACT) are recommended in DRC [7] for their proven efficacy and safety.

Since malaria episodes start at the household, making correct early decisions is key: Children with signs of potential malaria infection should see the Community Health Worker (CHW) where available, or be presented with no delay to a Health Centre. Instead, some patients or parents of sick children will resort to self-medication which is common in Africa and the DRC [8,9] or revert to traditional or unconventional treatments depending on the information available in the household, the perceived urgency of the situation, cultural and religious beliefs and economic considerations [10,11].

Understanding the knowledge, and the resulting behaviour of households in case of suspected malaria and their determinants will help to formulate better adapted and practicable strategies to guide patients and to reduce the number of fatalities in DRC.

## 2. Materials and Methods

### 2.1. Health Area Selection

At operational level, the health system of the DRC is composed of 517 Health Zones (Zone de Santé), each of them served by at least one General Referral Hospital. Each Health zone is composed of several Health Areas (Aires de Santé) in which Health Centres (Centres de Santé) offer integrated health services for the local population. We carried out in 2014 a study on rational use of antimalarials in health facilities (the URAP study = Usage Rationnel des AntiPaludiques) in all the 11 provinces of the DRC [12]. DRC had not yet transitioned from 11 to 26 provinces at that time. In each province, one General Referral Hospital (GRH), one Rural Health Centre (RHC) and one Urban Health Centre (UHC) were selected for participation. The current study presented here, assessing the use of antimalarials at community level was carried out in the health areas of rural and urban health zones selected in the URAP study. GRH were not considered since their population is covered by the different health centres of their Health Zone. The ensemble covers a catchment area of approximately 3 million inhabitants according to the data collected in Health Centres during the survey.

### 2.2. Household Selection

All households in the selected regions having had a case of malaria that presented to a participating Health Centre in the two weeks preceding the survey were selected. The addresses of these patients were extracted, and the listed households visited by the study teams.

### 2.3. Data Collection

A questionnaire was developed, and pre-tested in 20 households in the Mont Amba Health Zone in Kinshasa. After this validation, the questionnaire was administered by investigators to the patient or caregiver of a sick child. Data were collected in March and April 2018. The following parameters were collected: Household size, age of the responder, education level of the responder, level of knowledge about antimalarials, and behaviour in case of a suspected malaria episode.

Assessment of knowledge about antimalarials was made in four steps: First, we sought to know if the responder had received information on malaria treatment; second, we asked the main source of this information; third, we asked if the drugs recommended for the management of malaria were known, and fourth, we verified this knowledge by asking to cite these drugs. If the responder was able to name at least one recommended antimalarial drug without mentioning a non-recommended drug, the answer classified the responder as knowing the antimalarials.

Behaviour was evaluated by asking what action was normally taken when malaria infection was suspected. Consulting the Community Health Worker, the Healthcare provider at the Health Centre or going to any health facility (public or private) for rapid Diagnostic Test (RDT) was considered as correct behaviour.

The data was entered using Epi-info 7, CDC Atlanta, then exported in Microsoft Excel 365 and analysed using Stata 14 software (StataCorp. 2015. Stata Statistical Software: Release 14. College Station, TX: StataCorp LP).

### 2.4. Statistical Analysis

Variables were described by count or frequencies. When necessary, χ2 square test was used to compare them. A *p* value < 0.05 was considered statistically significant. Logistic regression was used to assess determinants of the dependent variables, knowledge of antimalarials and good behaviour in case of suspected malaria. Age of the responder, sex, education level, location (urban or rural), religion, size of the household and source of information on antimalarials were considered as explanatory variables for knowledge of antimalarials. For good behaviour, the above-mentioned variables plus knowledge of the antimalarials were used as explanatory variables. After bivariate analyses, all variables with a statistical significance of 0.2 and below were included in the multivariate model. The final model was obtained by backward elimination.

### 2.5. Ethical Consideration

This study did not expose participants to more than minimal risk. Approval was obtained from the Ethics Committee of the Protestant University of Congo under number CEUP 0048. All responders provided written informed consent; parents or legal representatives provided informed consent for minor responders. Confidentiality of their information is maintained: source data are only accessible to study team and kept in locked area. The data analysed did not include individual identifiers.

## 3. Results

All households with one or more cases of malaria during the previous 15 days were offered the possibility to participate in the study. A number of 1732 households participated in the study.

### 3.1. Characteristics of the Households

More than half of the responders (53.2%) lived in rural area; the median age was 37 years (6–98 years). Households were composed of an average of 7.1 members (CI 6.9–7.2). Half of the households (829/1700) were composed of 6–10 persons; 36.7% of them had less than six persons and 14.6% more than 10 persons. About 3.5% (19/1668) of the responders were minors. Regarding education, 57.8% of the responders (981/1699) were educated up to the secondary school; 18.8% up to primary school, 16.5% to university and 6.4% were illiterate. In terms of religion, 34.5% (573/1663) of the responders were Protestants, 33.0% were Catholic, 23.8% Evangelical Christians, 5.3% Muslims, 2.5% Kimbanguists. Less than 1% were followers of African religions.

Most of the responders (1060/1721 = 61.6%) had been informed about malaria treatment. The main source of that information was healthcare professionals of the Health Centres as shown in Table 1. The next source of information was media (radio, or television), followed by Community Health Workers (CHWs). Surprisingly, less than 1% received information from pharmacies, schools, and churches, which are places that are very often frequented in DRC. Most of the households (58.6%) used self-medication in case of suspected malaria; 37.9% went to the Health Facilities, whereas 3% used herbal medicines. None of the responders reported seeking health services from Community Health Workers.

### 3.2. Knowledge of Recommended Antimalarials

Nearly two third of the responders (61.6%) had been informed about recommended antimalarials; 1059 responders named what they considered as recommended antimalarials and among them, 742 (70.1%) were able to name correctly at least one recommended antimalarial.

Table 2 shows that the proportion of responders knowing the recommended antimalarial is comparable in urban and rural area, in the different age groups, gender, size of the households, religion, and source of information on antimalarials. However, the proportion increases with the level of education. 

As shown in Table 3, university- and secondary school-educated responders were more likely to know recommended antimalarials compared to illiterate (OR = 2.50 (1.04–6.00), *p* = 0.040, for university and 2.27 (1.00–5.15), *p* = 0.049 for secondary school). Responders from larger households were less likely to know recommended antimalarials; especially those from households with more than 10 persons (OR = 0.63 (0.41–0.98), *p* = 0.039). 

Having received information on recommended antimalarials was not a determinant of their knowledge. However, one source of information is notably effective: the media (radio and television). Those who received information from the media were more likely to know recommended antimalarials compared to those who received information from medical staff of the Health Centres: OR = 1.46 (1.01–2.10), *p* = 0.042.

### 3.3. Behaviour in Case of Suspected Malaria 

In case of a suspected malaria, most of the responders (58.6%) used self-medication; 37.8% went to the health facility for treatment and 3.0% used herbal medicines. As shown in Table 4, the proportion of responders with recommended behaviour is greater in those educated for more than 6 years (secondary school and university), those who knew recommended antimalarials and those who got information from the media. In terms of religion, more Catholics and Kimbanguist adopt the recommended behaviour. In multivariate regression, however, the predictors of good behaviour are religion of the responder and size of the household. Catholics are more likely to adopt recommended behaviour compared to Protestant (aOR = 1.56 (1.09–2.25), *p* = 0.016) and responders from larger households (>10 persons) are less likely to adopt recommended behaviour (aOR = 0.60 (0.37–0.98), *p* = 0.042). There may be an impact of education and knowledge of antimalarial, however differences are not statistically significant (*p* = 0.067). In terms of education, university responders behave comparably to illiterate responders, while primary and secondary school responders seem to be less likely to adopt the recommended attitude compared to illiterate responders, but differences are not statistically significant, as shown in Table 5. Source of information on antimalarials is not a predictor of recommended behaviour.

## 4. Discussion

This study focused on households in the communities served by the health centres described our previous study [12]. We investigated the determinants of knowledge of antimalarials and behaviour towards a suspected malaria case.

A total of 63.4% of the households were composed of more than six persons and the average size of household was 7.1 persons. This is greater than the average household size in DRC (5.3 persons) according to the last Health and Demographic Surveillance (HDS) [5]. The larger household size in our study may be due to targeting households with malaria cases. In facts, some studies, including in DRC, have shown that larger households are at increased risk of malaria [13,14,15]; this may have resulted in overrepresentation of larger households in our sample. Most importantly, size of the households should be taken in account in the malaria control activities because, being part of a large household (>10 people) significantly decreased the likelihood of knowing the recommended antimalarials and adopting recommended behaviour. Larger families are also more likely to resort to self-medication [16]. A study in the DRC showed that self-medication mostly concerns antimalarials, and that the users usually do not know the exact dosage of the drug used and do not check the expiry date [8]. In addition, self-medication may result in harmful outcomes, such as treatment failure, avoidable adverse events and even death [17]. Self-medication is also part of inappropriate use of antimicrobials which is the main driver of antimicrobial resistance [18,19], one of the 10 threats to public health in the 21st century [19]. Drugs used in self-medication come from pharmacies, leftover from previous malaria episodes, neighbours and non-official drug sellers [20]. This is another setback, since non-official drug sellers do not handle medicines appropriately, especially in DRC where some of them have confirmed selling medicine that is expired or has been banned by the Congolese government [21]. Since larger households are, at the same time, at increased risk of malaria [4,5,6] and prone to ignorance and unrecommended behaviour, special attention needs to be paid to them and ways must be found to help them be involved in the fight against Malaria. CHWs and other health workers could target these families and support them in the fight against malaria. This assumes the capacitation of these agents to improve their performance.

Only 61.6% of the responders (1060/1721) said they had been informed about recommended antimalarial drugs. In addition, 742 out of the 1059 responders who tried to cite them were able to name at least one recommended antimalarial. This represents 70.7% of those who answered to the question and 42.8% of all responders. These figures suggest that 40% of the population has not heard of antimalarials and that 30% cannot name at least one drug against malaria, which is still the leading cause of mortality in DRC [5,22,23], killing more than 40,000 of people, mostly children every year in the country [1]. In order to tackle this concerning level of ignorance, measures should be put in place to ensure better knowledge of the recommended drugs. The most effective ways should be used to communicate that information to the population and to make sure the information is suitable for the audience: having received information on antimalarials failed to be a predictor of their knowledge. However, knowledge of antimalarials was better when the responder was more educated, came from a smaller household, and had received information from the media. Multivariate regression shows that university or high school education is a statistically significant predictor of better knowledge of antimalarials. Primary school is not a predictor of knowledge and may reduce the likelihood of adopting recommended attitude. This is in line with a study in Nigeria that found completion of only basic education to be associated with a reduction in the likelihood of seeking healthcare from formal sources [24]. A higher level of education indeed allows a better understanding of the message provided, not only by the medical staff of the Health Centre (HC), which is the most prevalent source of information, but also from the media and other sources of information. However, not all of population is highly educated. In our study, 6.6% of the responders were illiterate and 18.9% had only basic education in primary school. Since this population is likely to be concerned by malaria, messages adapted to them should also be provided. Such adaptation should include not only the information tools (image boxes), but also the language used. In the context of the DRC, where French is the official and teaching language, while the national languages (Lingala, Swahili, Kikongo, or Tshiluba) are used for daily communication, language is crucial: medical doctors and all medical staff are educated for years in French and because of this, they tend to use it for daily communication including with patients. This may create a language barrier that may explain why the principal source of information for almost half of the respondents (medical staff of the HC) fails to be the best determinant of knowledge of malaria drugs. Indeed, there may be language barriers in communication between medical staff and patients [25,26], especially those from villages, in poor countries [27] such as DRC.

The second and the best source of information on antimalarials is the mass media, especially radio and television. This source is a significant predictor of best knowledge of antimalarials. Indeed, mass media plays an important role in healthcare communication in the 21st century [16,28]. The media reaches many persons at a time. Moreover, the language is simpler, associated with images and performances of famous comedians, musicians, film actors and other celebrities. This catches attention and facilitate comprehension of the message. Since the media is an important and more effective source of information on malaria treatment, it should be used more, and the message conveyed should be diversified. Social media, which is increasingly taking up space alongside traditional media [29] should also be used and could produce results comparable if not superior to traditional media.

There is also a need to communicate malaria information in churches and other religious meeting places. The fact that only two persons received the information from the churches suggests that the information on malaria is not sufficiently conveyed in these places. Since the population of the DRC is known to be deeply religious [30] and because religion has an influence on households’ practice regarding malaria [31], churches would be a good place to spread messages about antimalarials. In addition, religion had an influence on the behaviour of our responders. For example, belonging to evangelical churches in which miracle healing is one of the main teachings, may decrease by 20% the likelihood of adopting the recommended behaviour in case of suspected malaria, but the decrease was not statistically significant. On the other hand, Catholic religion, which teaches less about miracle healing, is a statistically significant predictor of recommended attitudes; the same has been shown in another study in Nigeria [32]. Nevertheless, giving message on malaria and its treatment in churches could be a way to reach a great number of Congolese and help them know better for better behaviour.

Another source of information that needs attention is community health workers (CHWs). They are the third source of information for families and their importance has been described in several studies. They are considered to be the backbone of the elimination of malaria [33]; their involvement in integrated Community Management of Malaria (iCCM) has produced positive results in terms of early and appropriate treatment of Malaria [34]. Since CHWs typically reside in the community they serve, they have the unique ability to bring information where it is needed most. They can reach community residents where they live, eat, play, work, and worship [35]. Hence, CHWs can be crucial for adequate health seeking behaviour, and they are predestined to give advice on treatment adherence and about the risks of self-treatment. However, none of the responders reported seeking health services from CHWs in case of suspected malaria. CHWs are supposed to provide healthcare services concerning malaria, acute respiratory infections, and diarrhea to populations in the most remote areas, but our responders did not mention using their services. This could mean insufficient communication about the services provided by community health workers or proximity of our responders to other health facilities. CHWs are present and active in the selected communities since they are mentioned as the third source of information on antimalarials, but their intervention does not seem to be effective or noted as relevant. The likelihood of knowing antimalarials and adopting recommended behaviour was found to be diminished by 20% and 30%, respectively, when the information came from the CHWs compared to medical staff of the HC. The decrease is not statistically significant, but this may mean that there need to be a reflection on the role of CHWs in communication of information on malaria treatment and their education. Another study in the DRC showed their importance in reducing the prevalence of malaria in a rural area, but nevertheless saw the difficulty of perpetuating their activity [36]. CHWs usually come from low socio-economic background [37], and most of them work on a voluntary basis [35,37]. Adequate training and appropriate motivation and incentives may improve their performances. This may include provision of uniforms or badges for identification, bicycles or funding for transport, formal job descriptions and performance evaluation tools [38].

In summary, it is of paramount importance to improve knowledge of antimalarial drugs in the population. Other studies have shown that knowledge of malaria to be a determinant of health seeking behaviour [39,40,41], but in our study, statistical signification was not attained (*p* = 0.067).

## 5. Conclusions

DRC has the second highest morbidity and mortality rates related to malaria worldwide. The level of knowledge and attitude of the communities towards malaria cases suggest that DRC is set to be losing out: The level of knowledge among communities is too low for a disease as widespread and deadly as malaria. In addition, most households do not adopt the recommended behaviour towards a suspected malaria case. There is a need for more effective communication, and this includes empowering CHWs, adaptation of the message provided by medical staff and usage of more effective ways such as via the media, social media, and churches. Considering the high attendance of churches in DRC, communication about malaria in this place may be highly efficient. Special attention is needed for larger families, which are more affected by malaria and more prone to non-recommended attitudes. An emphasis on education, not simply basic but at least at the secondary level is necessary to better understand information about malaria and behave better. This enforces the mandatory multisectoral nature of the fight against malaria, which should include socioeconomic aspects and education.

## Figures and Tables

**Table 1:** Characteristics of the households.

Variable	*N*	Percentage
Location of the responders (n = 1554)		
Rural area	826	53.2%
Urban area	728	46.8%
Age of the responders (n = 1668)		
<18 y	59	3.5%
18–50 y	1251	75.0%
51–64 y	278	16.7%
65 y	81	4.9%
Gender of the responders (n = 1700)		
F	981	57.7%
M	719	42.3%
Education level of the head responders (n = 1699)		
Illiterate	111	6.5%
Primary	318	18.7%
Secondary school	981	57.8%
University	286	16.9%
Religion of the responders (n = 1662)		
Protestant	573	34.5%
Catholic	549	33.0%
Evangelical Christian	396	23.8%
Kimbanguist	42	2.5%
Muslim	88	5.3%
African religion	8	0.5%
Atheist	6	0.4%
Size of the household (n = 1700)		
<6 persons	623	36.7%
6–10 persons	829	48.8%
>10 persons	248	14.6%
Informed about treatment of malaria (n = 1721)		
No	661	38.4%
Yes	1060	61.6%
Know recommended malaria drugs (n = 1059)		
No	317	29.9%
Yes	742	70.1%
Main source of information on malaria drugs(n = 1057)		
Staff of the Health Centre	496	46.9%
Media	311	29.4%
Community Health Workers	132	12.9%
Relatives	84	7.9%
Pharmacy	3	0.3%
Other	27	2.6%
Training	4	0.4%
Attitude in case of suspected malaria (n = 1699)		
Self-medication	995	58.6%
Consultation to Health facility	643	37.8%
Use of Herbal medicine	51	3.0%
Other	9	0.5%
Malaria RDT in pharmacy	1	0.1%

RDT = Rapid Diagnostic test.

**Table 2:** Proportion of responders with knowledge of recommended antimalarials policy.

Variables	Know Recommended Antimalarials		*p* Value
	*N*	Percentage	
Location of the responder (*n* = 969)			
Rural area (*n* = 500)	352	70.4%	0.605
Urban area (*n* = 469)	323	68.9%	
Education of the responders/head of household (*n* = 1037)			
Illiterate (*n* = 34)	17	50.0%	0.010
Primary school (*n* = 164)	104	63.4%	
Secondary school (*n* = 638)	454	71.2%	
University (*n* = 201)	148	73.6%	
Age of the responders/head of household (*n* = 1032)			
≤18 y (*n* = 31)	24	77.4%	0.639
18–<50 y (*n* = 800)	567	70.9%	
50–<65 y (*n* = 161)	109	67.7%	
≥65 y (*n* = 40)	29	72.5%	
Gender of the responders/head of household (*n* = 1040)			
Female (*n* = 602)	414	68.8%	0.487
Male (*n* = 438)	310	70.8%	
Size of the household (*n* = 1042)			
<6 persons (*n* = 378)	277	73.3%	0.126
6–10 persons (*n* = 507)	349	68.8%	
>10 persons (*n* = 157)	102	65.0%	
Informed about antimalarials (*n* = 1055)			
No (*n* = 134)	94	70.1%	0.978
Yes (*n* = 921)	645	70.0%	
Religion (*n* = 1017)			
Protestant (*n* = 316)	227	71.8%	0.215
Catholic (*n* = 370)	269	72.7%	
Evangelical Christian (*n* = 231)	148	64.1%	
Kimbanguism (*n* = 32)	24	75.0%	
Islam (*n* = 61)	41	67.2%	
African religions (*n* = 4)	2	50.0%	
Atheist (*n* = 3)	3	100.0%	
Main source of information (*n* = 916)			
Healthcare professionals (*n* = 445)	304	68.3%	0.067
Media (*n* = 255)	195	76.5%	
CHW (*n* = 122)	78	63.9%	
Relatives (*n* = 65)	40	61.5%	
Pharmacy (*n* = 3)	2	66.7%	
Other (*n* = 22)	16	72.7%	
Training (*n* = 4)	4	100.0%	

CHW = Community health worker.

**Table 3:** Determinants of knowledge of recommended antimalarials.

Bivariate Regression				Multivariate Regression		
Variables	OR	95% CI	*p*	aOR	95% CI	*p*
Age of the responder						
6–18 y	1					
18–49 y	0.71	0.30–1.67	0.432			
50–64 y	0.61	0.247–1.51	0.286			
≥65 y	0.77	0.26–2.29	0.637			
Gender of the responder						
F	1					
M	1.09	0.84–1.44	0.488			
Education of the responder						
Illiterate	1			1		
Primary school	1.73	0.82–3.65	0.147	1.69	0.71–4.03	0.236
Secondary school	2.47	1.23–4.93	*0.011*	2.27	1.00–5.15	*0.049*
University	2.79	1.33–5.86	*0.007*	2.50	1.04–6.00	*0.040*
Religion of the responder						
Protestant	1					
Catholic	1.04	0.75–1.46	0.800			
Evangelical Christian	0.69	0.48–1.00	0.054			
Kimbanguist	1.17	0.51–2.71	0.704			
Muslim	0.80	0.45–1.44	0.467			
African religion	0.39	0.05–2.82	0.353			
Size of the household						
˂6 members	1			1		
6–10 members	0.80	0.59–1.08	0.151	0.82	0.59–1.13	0.219
≥10 members	0.68	0.45–1.00	0.055	0.63	0.41–0.98	*0.039*
Informed about antimalarials						
No	1					
Yes	0.99	0.67–1.48	0.978			
Main source of information on antimalarials						
Medical staff	1			1		
Media	1.51	1.06–2.14	*0.022*	1.46	1.01–2.10	*0.042*
CHW	0.822	0.54–1.25	0.361	0.84	0.55–1.30	0.444
Relatives	0.74	0.43–1.27	0.277	0.81	0.47–1.42	0.470
Pharmacy	0.93	0.08–10.31	0.951	0.93	0.08–10.41	0.953
Other	1.24	0.47–3.23	0.664	1.28	0.48–3.35	0.622

CHW = Community health worker.

**Table 4:** Proportion of responders with recommended behaviour in case of suspected malaria.

Variable	Recommended Behaviour		*p* Value
	*N*	Percentage	
Education of the responders (*n* = 1666)			
Illiterate (*n* = 110)	34	30.9%	0.001
Primary school (*n* = 312)	95	30.4%	
Secondary school (*n* = 963)	368	38.2%	
University (*n* = 281)	129	45.9%	
Age of the responders (*n* = 1636)			
≤18 y (*n* = 58)	21	36.2%	0.523
18–<50 y (*n* = 1228)	452	36.8%	
50–<65 y (*n* = 271)	107	39.5%	
≥65 y (*n* = 79)	29	36.7%	
Gender of the responders (*n* = 1671)			
Female (*n* = 966)	353	36.5%	0.229
Male (*n* = 705)	278	39.4%	
Size of the household (*n* = 1671)			
<6 persons (*n* = 614)	239	38.9%	0.559
6–10 persons (*n* = 814)	310	38.1%	
>10 persons (*n* = 243)	85	35.0%	
Informed about antimalarials (*n* = 1690)			
No (*n* = 644)	238	36.9%	0.544
Yes (*n* = 1046)	402	38.4%	
Know antimalarials (*n* = 1046)			
No (*n* = 315)	96	30.5%	0.001
Yes (*n* = 731)	299	40.9%	
Religion of the responders (*n* = 1632)			
Protestant (*n* = 562)	211	37.5%	0.065
Catholic (*n* = 540)	220	40.7%	
Bible Christian (*n* = 386)	132	33.9%	
Kimbanguism (*n* = 42)	23	54.8%	
Islam (*n* = 88)	26	29.5%	
African religions (*n* = 8)	4	50.0%	
Atheist (*n* = 6)	3	50.0%	
Source of information (*n* = 1045)			
Medical staff (*n* = 490)	191	39.0%	0.269
Media (*n* = 307)	127	41.4%	
CHW (*n* = 131)	42	32.1%	
Relatives (*n* = 83)	27	32.5%	
Pharmacy (*n* = 3)	1	33.3%	
Other (*n* = 27)	12	44.4%	
Training (*n* = 4)	0	0.0%	

CHW = Community health worker.

**Table 5:** Determinants of recommended behaviour.

Variable	OR	95% CI	*p*	aOR	95% CI	*p*
Age of the responder						
6–18 y	1					
18–49 y	1.03	0.59–1.78	0.926			
50–64 y	1.15	0.64–2.07	0.642			
65 y and above	1.02	0.50–2.07	0.952			
Gender of the responder						
F	1					
M	1.13	0.93–1.38	0.229			
Education of the responder						
Illiterate	1					
Primary school	0.98	0.61–1.57	0.928	0.37	0.13–1.04	0.059
Secondary school	1.38	0.90–2.11	0.135	0.58	0.22–1.52	0.27
University	1.90	1.19–3.03	*0.007*	1.03	0.38–2.83	0.95
Religion of the responder						
Protestant	1					
Catholic	1.14	0.90–1.46	0.277	1.56	1.09–2.25	0.016
Evangelical Christian	0.86	0.66–1.13	0.292	0.80	0.51–1.24	0.313
Kimbanguist	2.01	1.07–3.78	*0.030*	2.12	0.92–4.89	0.077
Muslim	0.70	0.43–1.14	0.149	0.76	0.37–1.53	0.438
African religion	1.66	0.41–6.72	0.475	10.50	0.88–125.11	0.063
Atheist	1.66	0.33–8.32	0.535	0.61	0.05–7.68	0.703
Size of the household						
˂6 members	1					
6–10 members	0.97	0.78–1.20	0.746	0.81	0.58–1.12	0.204
≥10 members	0.84	0.62–1.15	0.283	0.60	0.37–0.98	*0.042*
Informed about antimalarials						
No	1					
Yes	1.06	0.87–1.30	0.544			
Know antimalarials						
No	1					
Yes	1.58	1.19–2.09	*0.001*	1.37	0.98–1.92	0.067
Source of information on antimalarials						
Medical staff	1					
Media	1.10	0.83–1.48	0.503	1.19	0.84–1.71	0.33
CHW	0.74	0.49–1.11	0.147	0.69	0.42–1.14	0.148
Relatives	0.75	0.46–1.24	0.264	0.67	0.35–1.28	0.223
Pharmacy	0.78	0.07–8.69	0.842	1.02	0.09–12.25	0.985
Other	1.25	0.57–2.73	0.572	1.25	0.46–3.40	0.66

CHW = Community health worker.

## Data Availability

All excel files are available from the Figshare database doi:10.6084/m9.figshare.14046101 (accessed on 22 August 2021).
